# The Influence of Web- Versus Paper-based Formats on the Assessment of Tobacco Dependence: Evaluating the Measurement Invariance of the Dimensions of Tobacco Dependence Scale

**DOI:** 10.4137/sart.s960

**Published:** 2009-02-17

**Authors:** Chris G. Richardson, Joy L. Johnson, Pamela A. Ratner, Bruno D. Zumbo

**Affiliations:** 1 School of Population and Public Health, University of British Columbia.; 2 Centre for Nursing and Health Behaviour Research, School of Nursing, University of British Columbia.; 3 Measurement, Evaluation and Research Methodology Program, University of British Columbia.

**Keywords:** tobacco dependence, adolescent, web-based survey, internet, psychometrics, validity, measurement invariance

## Abstract

The purpose of this study was to examine the influence of mode of administration (internet-based, web survey format versus pencil-and-paper format) on responses to the Dimensions of Tobacco Dependence Scale (DTDS). Responses from 1,484 adolescents that reported using tobacco (mean age 16 years) were examined; 354 (23.9%) participants completed a web-based version and 1,130 (76.1%) completed a paper-based version of the survey. Both surveys were completed in supervised classroom environments. Use of the web-based format was associated with significantly shorter completion times and a small but statistically significant increase in the number of missing responses. Tests of measurement invariance indicated that using a web-based mode of administration did not influence the psychometric functioning of the DTDS. There were no significant differences between the web- and paper-based groups’ ratings of the survey’s length, their question comprehension, and their response accuracy. Overall, the results of the study support the equivalence of scores obtained from web- and paper-based versions of the DTDS in secondary school settings.

The Dimensions of Tobacco Dependence Scale (DTDS)^1^ was developed in response to the recognized need for a multi-dimensional measure of tobacco dependence for the study of emerging tobacco dependence in adolescent populations.^2^,^3^ Based on our previous qualitative research of adolescents’ experiences of the “need to smoke”,^4^ we developed a preliminary set of 54 DTDS items that assessed various aspects of tobacco dependence as experienced by adolescents. An exploratory factor analysis of responses to the items was carried out using quantitative data gathered from a random sample of 513 adolescent smokers. The results indicated that the items represented four dimensions of tobacco dependence: social, emotional, sensory, and physical dependence characterized by nicotine addiction.^1^ Subsequent applications of non-parametric item response modeling and confirmatory factor analyses of data from a sample of 1,425 adolescent smokers led to the removal of several poorly discriminating items and confirmation of the four factor structure in the remaining 35 items.^5^ The next step in our ongoing validation of the DTDS was to examine the influence of a web- versus paper-based format on the psychometric performance of the DTDS.

Use of the World Wide Web (WWW) to gather survey data has the potential to revolutionize health behavior research, especially with adolescents who are much more likely than adults to respond positively to, and navigate efficiently through, electronically based tests. Web-based surveys (as distinguished from email surveys that typically consist of simple text-based messages) possess highly refined survey capabilities far beyond those of traditional pencil-and-paper survey approaches. For example, the use of a web-based survey facilitates the use of extensive and difficult skip patterns, “pop-up” instructions for specific items, “drop-down” boxes that provide extensive lists of response options, and countless possibilities for using colors, shapes and images. In addition to relatively more options for the design and presentation of survey content, web-based surveys eliminate the costly and time-consuming tasks of survey printing, distribution, and manual or electronic scanning for data entry. Privacy can be ensured through the use of personalized pass codes and data encryption technologies.

Although web-based surveys have tremendous potential, their use is accompanied by many new design and implementation considerations, including layout features, access to computers, the level of technical sophistication of respondents, and the compatibility of operating systems and screen configurations.^6^ For example, scrollable drop boxes containing lists of response options may be used instead of a series of radio buttons to save screen space. However, researchers have found that order effects related to primacy or visibility may be substantially magnified when drop boxes contain an initially visible subset of item responses (i.e. those items on display before scrolling down the remaining list).^7^ The availability of clarification features (e.g. hyperlinked definitions) represents another example of how what initially appears to be a major benefit may ultimately be of little use to respondents. For example, Conrad, Couper, Tourangeau, and Peytchev^8^ found that respondents’ use of clarification features (e.g. pop-up definitions) increased when activated through mouse roll over requests rather than click-based requests; however, the majority of respondents did not use the available definitions. They speculated that the extra effort associated with requesting a definition combined with the cognitive effort required to integrate the new knowledge discouraged use of most clarification features. Until researchers have a clear understanding of the effects of these innovations on survey responders, it may be best to continue to rely on the practical advice offered by Dillman, Tortora, and Bowker^9^ regarding question format. Specifically, Dillman et al. recommended: (a) the use of conventional formats in web-based surveys that closely matched paper questionnaire formats and (b) avoidance of question structures that have been problematic in paper-based questionnaires.

Issues related to sampling and differences in the accuracy of survey responses also have been the subject of investigation, especially in the addictions field. For example, researchers have reported finding no differences in rates of self-disclosed illicit drug use^10^ and rates of secondary consequences associated with substance use^11^ between self-administered questionnaires completed via web-based surveys versus mailed-out paper-based surveys. However, investigations involving other types of personal disclosure have found that using a computer-based survey is associated with greater levels of self-disclosure.^12^,^13^

Although research has been carried out on the influence of web-based surveys on the disclosure of personal information, the influence of a web-based survey format on the psychometric functioning of measurement tools appears to have been rarely discussed. Researchers have shown that converting a measure from a paper-and-pencil version to a computer- or web-based version can change the way respondents perceive and respond to the measure;^14^ however, little is known about how these format changes affect the reliability and internal validity of measurement instruments. Although research about the nature and extent of differences between web- and paper-based modes of survey administration is still in its infancy, experts in psychological measurement agree that the equivalence of these two different modes of survey administration cannot be assumed, but rather must be demonstrated for each instrument.^15^ McCoy et al.^14^ noted that the situation was best characterized by Cronbach,^16^ “It seems that the conventional and computer versions of a survey do usually measure the same variables, but difficulty or reliability can easily change. Whether the computer version is ‘the same survey’ must be questioned with each instrument in turn psychologically” (p. 48).

It is therefore not surprising that the Standards for Educational and Psychological Testing e.g. standard 4.10,^17^ require that a validation study be conducted to ensure the equivalence of web-based and paper-and-pencil versions of instruments. One method of establishing the equivalence of web and paper formats is through the testing of measurement invariance. Although many instruments have been subjected to tests of measurement invariance across groups based on gender, race/ethnicity, language and age, to the best of our knowledge this study represents the first application of this technique to the assessment of web versus paper modes of a tobacco dependence measure.

## Measurement invariance

Measurement invariance refers to “whether or not, under different conditions of observing and studying phenomena, measurement operations yield measures of the same attribute”.^18^ In the context of this investigation, the different conditions are defined by assessments made using web-based and paper-based versions of the DTDS. If researchers are unable to establish measurement invariance, then they cannot be confident that observed differences in mean scores are due to true differences between groups, on the construct of interest, or if the differences are due to systematic biases in the way people respond to items presented in the different survey formats (i.e. web- versus paper-based). 19 Differences in relationships among various scale scores might be the result of real differences in structural relations between constructs, scaling artifacts, differences in scale reliability, or in the most extreme case, actual nonequivalence of the constructs being assessed (Steenkamp and Baumgartner).

Multi-group confirmatory factor analysis^20^ represents one of the most powerful and versatile approaches to assessing scale-level measurement invariance.^19^ In this approach, the assessment of measurement invariance involves comparing the statistical fit of successively more constrained multi-group structural equation models associated with each level of invariance. For example, to test for metric invariance, the researcher determines whether the statistical fit of the more constrained metric invariance model (factor loadings held equal across groups) is worse than the same model with no constraints on the values of the factor loadings for the two groups. The following summary outlines the specific tests of invariance examined in this study, which were adapted from the recommendations of Steenkamp and Baumgartner.

### Configural invariance

The configural invariance approach is based on the principle of simple structure^21^ and implies that the items comprising the measurement instrument should exhibit the same configuration of salient (nonzero) and nonsalient (zero or near zero) factor loadings across different groups.^18^ Although the same items should load onto the same latent factors across groups, the size of the loadings may differ at this level of invariance. If the same items do not load onto the same factors across groups, then the constructs being assessed should not be treated as equivalent; that is, the latent constructs are not the same.

### Metric invariance

Although configural invariance indicates that the same items are related to the same latent variables across groups, it does not imply that respondents in different groups respond to the items in a way that supports the meaningful comparison of ratings across groups. Metric invariance is stricter than configural invariance in that it introduces the concept of equal metrics or scale intervals across groups.^22^ If an item satisfies the requirement of metric invariance, scores on the items can be meaningfully compared across groups. Because the factor loadings carry the information about how changes in latent scores relate to changes in observed scores, metric invariance is tested by constraining the item loadings to be equivalent across groups.

### Scalar invariance

Configural and metric invariance require only information about the covariation of the items in different groups. However, in many situations it is important to conduct mean comparisons across groups. In order for such comparisons to be meaningful, scalar invariance of the items is required.^23^ Scalar invariance implies that cross-group differences in the means of the observed items are due to differences in the means of the underlying construct(s). Even if an item measures the latent variable with equivalent metrics in web- and paper-based groups (i.e. metric invariance has been established), scores on that item can still be systematically biased upward or downward if the item intercepts are different for the two groups. Comparisons of observed group means using items biased in this way are meaningless unless the bias is removed from the data (Meredith).

### Error variance invariance

The last form of invariance tested in this study was error variance invariance. If items are metrically invariant and the error variances are invariant, then the items are equally reliable across groups.

Despite the advantages of this approach to testing measurement invariance, recent research suggests that analyses of rating scale data using the methods described by Steenkamp and Baumgartner^19^ may not detect item bias when analyzing a covariance matrix using maximum likelihood estimation. 24,25 One method of addressing this limitation is to perform an item-level analysis of differential item functioning.

## Study objectives

The primary objective of this investigation was to examine the influence of a web- versus paper-based mode of administration on the psychometric performance of the DTDS.

Specifically, we compared responses to a web-based version of the DTDS with responses from a paper-based version to determine if there were: (a) differences in data quality in terms of missing data or item omissions and (b) differences in the psychometric characteristics of responses to the DTDS items in terms of measurement invariance. In addition, we tested the following secondary hypotheses: (c) there would be no difference in self-reported ratings of questionnaire length, comprehension and accuracy of responses and (d) there would be no differences in the length of time respondents needed to complete the survey.

## Method

### Participants and sampling

The data analyzed in this study were obtained from the British Columbia Youth Survey on Smoking and Health 2 (BCYSSH2), which was conducted between March and June 2004. In addition to the DTDS items, the survey included questions addressing a wide range of tobacco, drug and health-related behavior. The survey was conducted in 49 secondary schools located in regional school districts that were previously found to have higher than average rates of tobacco use in the province of British Columbia, Canada (i.e. outside the densely populated urban area of Vancouver).^26^ The selection of students for inclusion varied across the 49 schools. The entire student body or all students in a particular grade were recruited for participation in 22 schools with the remaining 27 schools selectively recruiting students. The selective recruitment was carried out by enrolling students in courses taken by most students (e.g. all students in Grade 9 Career and Personal Planning). Of approximately 10,000 potential respondents, 8,225 completed the questionnaire. Non-response was primarily due to students being absent from school; however, 76 students refused to participate despite being present in the classroom where the questionnaire was administered. The average response rate across schools was 84%.

### Implementation of web-based survey

**Figure 1:**
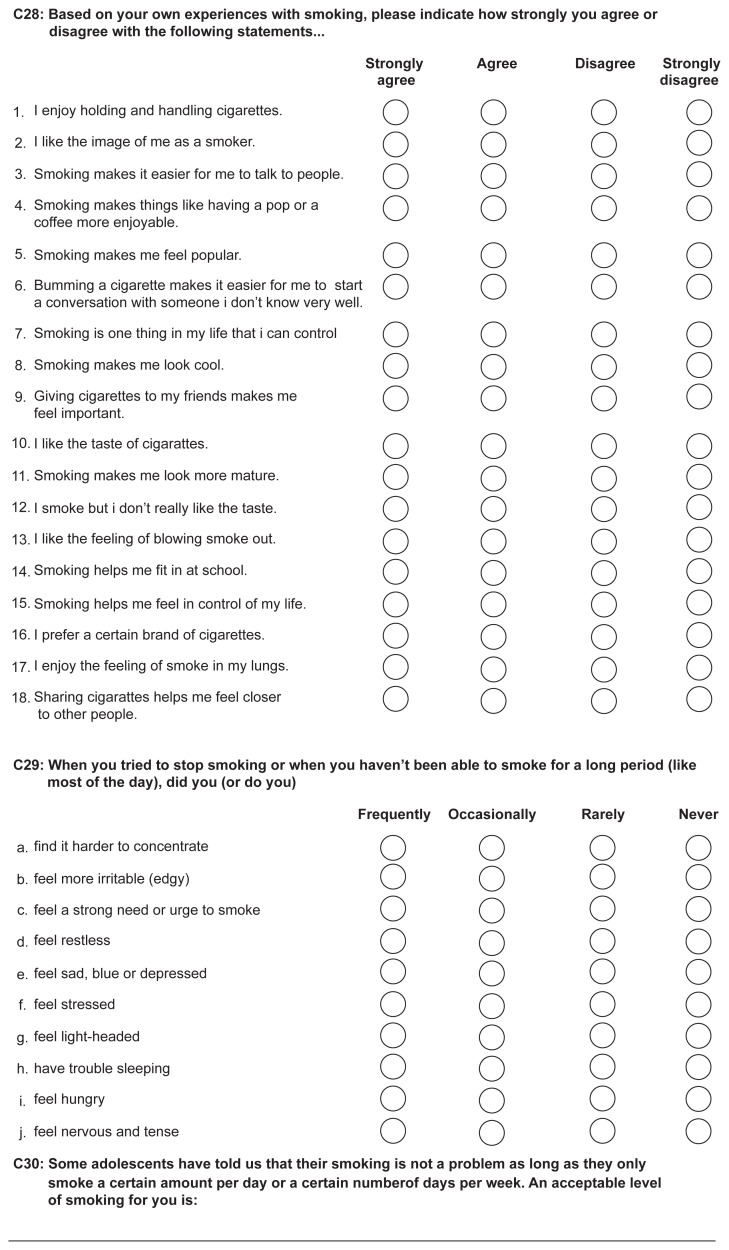
Sample page from the paper-based version of the BCYSOSH2 containing DTDS questions. The survey was printed in black and white on legal sized paper and respondents were instructed to indicate their response using either a checkmark or an “X”.

**Figure 2:**
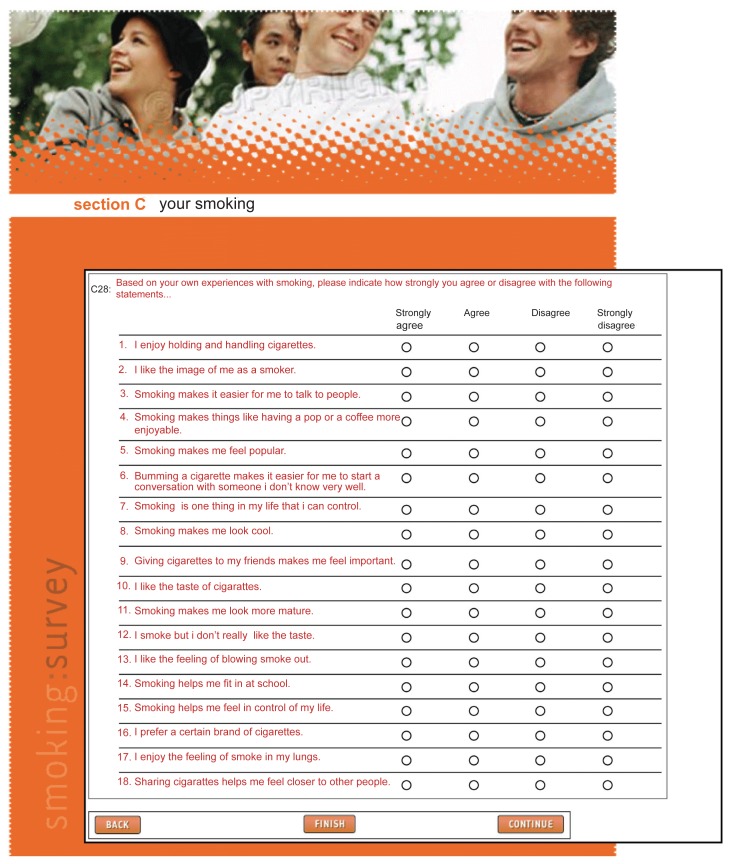
Snapshot from the web-based version of the BCYSOSH2 containing DTDS questions. Respondents “clicked” on a radio-button to indicate their response for each question.

Although randomization of classes in the schools to survey mode was intended, it became apparent that many schools had inadequate computer facilities to manage web-based questionnaire administration to whole classes. Although several classes were initially randomly assigned to and subsequently completed a web-based survey, the allocation to survey-type (web verses paper) was primarily based on the availability of time and space in computer labs. In both scenarios, the questionnaires were completed in a classroom or similar setting with trained research staff available to monitor the students and to answer their questions. The web-based questionnaire was designed by professional web designers, included color graphics, and was hosted on a web server maintained by the survey designers. The two versions of the survey were identical in terms of question content and question order. The paper-based questionnaire was printed on legal sized paper and included section headings to identify groups of items and several graphics to break up the text. The web-based questionnaire included a graphic and section heading at the top of each webpage (see Fig. 1 and 2 for examples of the paper- and web-based layouts).

The presentation of the DTDS question and response options were similar in the web- and paper-based formats. Radio-buttons for the item response options were used in the web-based survey. There were two major skip patterns presented to accommodate the responses of tobacco smokers and non-smokers. In the paper version, printed instructions indicated to which page and question the respondent should skip. The skip patterns in the web-survey were programmed such that the respondents were not exposed to irrelevant questions. At the bottom of each webpage, the respondents were required to click on a response indicating that they wanted to return to the previous page, finish the survey, or continue to the next page. At the end of the web survey, the respondents were provided a list of incomplete items and were directed to return to the relevant pages, which required that they click on the reported page number; they also had the option of submitting the survey with missing responses. At the end of the paper-based survey, the respondents were advised to review their work and to ensure that all relevant questions were completed. The web-based survey automatically timed the students’ work and the paper-based questionnaire required that respondents record the time when they started and finished the questionnaire.

### Instruments

The questionnaire included several scales and items related to tobacco and marijuana use, physical and mental health status, life satisfaction, and sociodemographics. Those relevant to the current analysis are described below.

#### Family financial situation

A single item asked respondents, “*How would you describe your household’s financial situation* (*how much money your family has)?*” with the response options being: very well-off; well-off; a little above average; average; a little below average; below average; and poor.

#### Mother’s education

Respondents were asked to check the highest level of education completed by their mother from the following list: elementary school (up to Grade 8); some high school; high school; trades certification (i.e. carpenter, plumber); some community college/university; community college; university undergraduate degree (i.e. Bachelor of Arts, Bachelor of Science, etc.); Master’s degree (i.e. MA, M.Sc., MBA, MSW, etc.); and post graduate training/professional degree (i.e. M.D., Law, Ph.D., etc.). Responses were collapsed into 3 categories: Less than high school; Completed high school; Completed a post-secondary degree.

#### Father’s education

Respondents were asked to check the highest level of education completed by their father from the following list: elementary school (up to Grade 8); some high school; high school; trades certification (i.e. carpenter, plumber); some community college/university; community college; university undergraduate degree (i.e. Bachelor of Arts, Bachelor of Science, etc.); Master’s degree (i.e. MA, M.Sc., MBA, MSW, etc.); and post graduate training/professional degree (i.e. M.D., Law, Ph.D., etc.). Responses were collapsed into 3 categories: Less than high school; Completed high school; Completed a post-secondary degree.

#### The Dimensions of Tobacco Dependence Scale (DTDS)

The 35-item DTDS (see Table 1) was developed to assess four dimensions of emerging tobacco dependence: social dependence (6 items), emotional dependence (5 items), sensory dependence (5 items), and physical dependence characterized by nicotine addiction (19 items). Each of the items is answered via a 4-point scale coded as either “strongly agree (4), agree (3), disagree (2) or strongly disagree (1)” or “always (4), often (3), sometimes (2) or never (1).” Responses to each set of items are summed to create dimension-specific scores. The scale was specifically designed for use in adolescent populations and has been shown to provide valid and reliable measures of tobacco dependence in adolescents.^5^

#### Self reported accuracy of survey responses

As part of the survey evaluation, respondents were asked, “On the whole, how accurate were your answers in this survey?” The response options were: “very accurate, mostly accurate, mostly inaccurate, and very inaccurate.”

#### Self reported comprehension of survey questions

As part of the survey evaluation, respondents were asked, “Did you have difficulty understanding any of the questions?” The response options were: “understood all questions, difficulty understanding a few, and difficulty understanding many.”

#### Ratings of questionnaire length

As part of the survey evaluation, respondents were asked, “How did you find the length of this questionnaire?” The response options were: “much too long, a bit too long, about right, a bit too short, and much too short.”

### Analyses

#### Multi-group confirmatory factor analysis

Following the recommendations of Steenkamp and Baumgartner^19^ and Byrne,^27^ multi-group confirmatory factor analysis with maximum likelihood estimation was used to assess the measurement invariance of the responses to the two administration modes of the DTDS. Under this approach, the assessment of measurement invariance involves comparing the statistical fit of successively more constrained structural equation models associated with each level of invariance. The χ^2^ and χ^2^-difference tests, and several supplementary goodness-of-fit indices, are reported with the caveat that the relatively large sample size resulted in highly statistically powered χ^2^ and χ^2^-difference tests.

Given the sensitivity of the χ^2^ and χ^2^-difference tests, the following criteria, recommended by Hu and Bentler,^28^ were used to determine if there was a relatively good fit between the item covariances implied by the hypothesized measurement models and the item covariances observed in the data: a cutoff value close to 0.95 for the comparative fit index (CFI); a cutoff value close to 0.06 for the root mean square error of approximation (RMSEA); and a cutoff value close to 0.08 for the standardized root mean residual (SRMR). The CFI represents a measure of comparative fit that is essentially derived by comparing the model fit (i.e. model χ^2^) of the implied model with a baseline model that specifies no relationships between the variables included in the model. CFI scores range between 0 and 1, with higher numbers indicating a greater improvement in the fit of the implied model compared to the baseline model.^29^ The RMSEA and the SRMR are based on the analysis of residuals between the covariance matrix implied by the model and the covariance matrix observed in the data. The values of the RMSEA and SRMR range between 0 and 1 with lower values indicating better fit in terms of lower discrepancies between the model’s implied and observed covariances (Kelloway). More detailed information on the calculation and interpretation of these fit indices can be found in Hu and Bentler.^28^ Following the recommendations of Cheung and Rensvold,^30^ a change in the CFI ≤ –0.01 between successive levels of invariance was used as a cutoff within which invariance was not rejected. While cutoff criteria have been proposed for the other goodness of fit indices considered, the simulation studies by Cheung and Rensvold suggest that only the performance of the CFI can be considered to be independent of model parameters and sample size when testing for measurement invariance (the SRMR was not included in their simulations).

To avoid the list-wise deletion of cases with partial missing data, missing responses for DTDS items were imputed using the multiple imputation procedure in the software PRELIS 2.54.^31^ The software MPlus Version 3.1^32^ was used to conduct the tests of measurement invariance. Maximum likelihood estimation was used and a mean structure was specified for the multi-group structural equation models.

#### Differential item functioning

Following the methodology outlined by Zumbo,^33^ the item-level tests for DIF proceeded in a series of three steps that followed a natural hierarchy created by entering variables into successive ordinal regression models. The following modeling steps were repeated for each item in each of the DTDS dimension specific scales.

Step 1. The conditioning variable (e.g. the total score of the social dimension of the DTDS) was included in an ordinal logistic regression model predicting the response to the question item under investigation (e.g. response to item 1 of the social scale);
Step 2. In addition to the total score of the relevant dimension, the format variable (web- versus paper-based) was added to the ordinal logistic regression model predicting the response to the question item under investigation;
Step 3. In addition to the total score and the format variable, the interaction between total score and format was added to the ordinal logistic regression model predicting response to the question item under investigation.

After running the models described in each step, the χ^2^ and model R^2^ values associated with each step were used to compute the statistical tests for DIF for each item. The χ^2^ value for the final model including the interaction term (Step 3) was subtracted from the χ^2^ for the model that included only the conditioning variable (Step 1). The resulting χ^2^-difference was then compared to its distribution function with 2 degrees of freedom to determine the level of significance. This two-degrees of freedom χ^2^-test is a simultaneous test of uniform and non-uniform DIF.^33^

According to Zumbo,^33^ two criteria must be met to conclude that an item displays DIF: (a) the χ^2^-difference test (with 2 degrees of freedom) between Steps 1 and 3 must have a *p*-value ≤ 0.01 and (b) the corresponding measure of effect size represented by the change in model R^2^ between steps 1 and 3 must be ≥0.13. If an item is found to display DIF, the extent to which the DIF is uniform is determined by comparing the change in R^2^ values between the first and second steps (uniform DIF) with the change in R^2^ values between the first and third steps (uniform and non-uniform DIF). To examine the possible influence of the participants’ demographics and socio-economic status on format differences, all the models in the DIF analyses were run a second time with the following variables included as covariates: Age of participant in years, gender, self-reported family financial situation, mother’s highest level of education, and father’s highest level of education.

## Results

Of the 8,225 student surveys examined in this study, 11 did not answer the screening question regarding whether they had smoked in the past month. Of the remaining 8,214 cases, 1,484 (18.1%) indicated that they had smoked at least once in the month preceding the survey. Of these 1,484 smokers, 23.9% completed the web-based questionnaire and 76.1% completed the paper-based version.

The two groups of respondents were similar in terms of age and sex distribution. The sample of respondents who completed the web-based survey appeared to contain fewer experienced smokers (i.e. the web sample had a greater percentage of respondents in the lower categories of lifetime number of cigarettes smoked) who also had lower dimension-specific scores, on average, on the DTDS (see Table 2).

### Number of missing responses to the DTDS

Sixty-two percent of the respondents who answered the web format missed one or more of the 35 DTDS items, whereas 27% missed one or more items in the paper version. This difference was statistically significant (χ^2^_(1)_ = 142.7, *p <* 0.01). A review of the number of missing responses on an item by item basis indicated that the DTDS item, “I have strong cravings to smoke cigarettes” had a particularly poor response rate in the web survey (41% complete in the web format versus 91% in the paper format). The mean (95% confidence interval) and median number of missing responses to the 35-item DTDS were 4.5 (95% CI: 3.5, 5.5) and 1 in the web survey and 2.7 (95% CI: 2.2, 3.1) and 0 for the paper version of the survey, respectively. This difference was statistically significant (Mann-Whitney U = 136229.5, *p <* 0.01).

### Scale-level measurement invariance

The results of the multi-group structural equation model tests of successive levels of measurement invariance are presented in Table 4. The test of configural invariance, where the pattern of salient (nonzero) and nonsalient (zero) factor loadings was constrained to be equal for the web and paper groups failed the χ^2^ test (χ^2^_(1108)_ = 4226, *p <* 0.01), but demonstrated moderate to good fit on the CFI, RMSEA and SRMR according to the criteria of Hu and Bentler.^28^ We proceeded to assess the metric invariance of the DTDS by adding the constraint that the factor loadings for the indicators were equal for the two format groups. Although the χ^2^ increased slightly, the change was not significant (Δ χ^2^_(31)_ = 38, *p >* 0.05), the CFI was unaffected and the RMSEA and SRMR changed slightly. Based on the non significant change in χ^2^ and unchanged CFI, the DTDS was deemed to possess metric invariance and we proceeded to test for scalar invariance by adding the constraint that the item intercepts were equal in the web and paper groups. The fit of the resulting model was significantly worse according to the χ^2^ (Δ χ^2^_(31)_ = 126, *p <* 0.01), however the change in CFI of –0.003 was well below the cutoff of –0.01 recommended by Cheung and Rensvold^30^ and the RMSEA and SRMR indicated only a slight deterioration in model fit. The DTDS was thus deemed to possess scalar invariance and we proceeded to test for invariance in the item error variances by adding the constraint that they were equal in the web and paper groups. The resulting model had a significantly greater χ^2^ (Δ χ^2^_(35)_ = 275, *p <* 0.01), however the change in CFI of –0.006 was below the recommended cutoff of –0.01 and the RMSEA and SRMR indicated only a slight deterioration in fit. The DTDS was therefore considered to be invariant at the level of item error variances.

### Item-level measurement invariance

None of the DTDS items met Zumbo’s^33^ criteria for DIF (i.e. the χ^2^-difference test between Steps 1 and 3 must have a *p*-value ≤ 0.01 and the change in model R^2^ between steps 1 and 3 must be ≥0.13). Including the participants’ age in years, gender, self-reported family financial situation, mother’s highest level of education and father’s highest level of education, as covariates, did not alter the finding of no DIF.

### Ratings of survey accuracy, comprehension and length

The response frequencies for the ratings of survey accuracy, comprehension and length are presented in Table 3. There were no significant differences between the web- and paper-based formats in terms of self-reported accuracy of responses (χ^2^_(3)_ = 3.86, *p >* 0.05), self-reported comprehension of questions (χ^2^_(2)_ = 2.60, *p >* 0.05) and perceived length of questionnaire (χ^2^_(4)_ = 2.53, *p >* 0.05).

### Time required to complete full survey

The time required to complete the entire British Columbia Youth Survey on Smoking and Health 2 was significantly shorter in the web- (median = 40 minutes, mean = 40.6, s.d. = 12.5) compared with the paper-based version (median = 45 minutes, mean = 48.3, s.d. = 19.1) of the survey (t_(903)_ = 7.4, *p <* 0.01). The time for web survey completion was computed automatically, whereas respondents answering the paper version were asked to manually report a start and end time. Fifty-one percent of respondents in the paper-based group did not enter start and finish times on their surveys and were not included in the analysis of completion times.

## Discussion

The primary goal of this study was to investigate the impact of using a web- versus paper-based questionnaire format on the performance of the DTDS in adolescent smokers. Our results indicate that there were no differences in adolescent smokers’ evaluations of the perceived length of the entire survey, their comprehension of the survey, and ratings of the accuracy of their responses. Despite these consistencies, we found that the use of the web-based format was associated with a small but significant increase in the total number of missing responses, with one item, “I have strong cravings to smoke cigarettes,” in particular, demonstrating a particularly poor response rate in the web survey. This difference was surprising; given the lack of random assignment to questionnaire format, however, we can only speculate about the reasons for it.

An assessment of a potential order effect indicated that the “I have strong cravings to smoke cigarettes” item was viewed by the respondents within a group of other items (i.e. the item was not out of view or at the bottom of a list of items). Although the ages of the participants in the two format groups were similar, the percentage of respondents who reported their lifetime use of cigarettes as “a puff or a few puffs” was much greater in the web survey group (17.3% vs. 3.9% for the paper group). It is possible that respondents with little experience with tobacco smoking and its effects may have intentionally skipped some items (e.g. “I have strong cravings to smoke cigarettes”) because they could not relate to the content; however, we do not think such an effect would be restricted to a single item of a 35-item scale. It also is possible that the web format in some way facilitated the skipping of some items. However, this seems unlikely given that the appearance of the items was very similar across formats (e.g. the same groups of items appeared together on the screen as on each page of the paper version and no scrolling was required to see an item). Although it is possible that differing levels of missing data might bias our assessment of measurement invariance, we believe that the impact of this bias would be relatively minor given that the increased level of non-response was primarily related to a single item.

In addition to assessing the influence of survey format on the respondents’ overall evaluation of the survey and the extent of missing data, we examined the measurement invariance of the DTDS at the scale and item levels. The results of our invariance testing indicated that there were several statistically significant changes in the model χ^2^ that suggested a lack of measurement invariance (e.g. in the tests of configural and error variance invariances). However, the large sample size in this study is associated with highly powered χ^2^ and χ^2^-difference tests, which can identify small statistically significant differences that ultimately have very limited meaning in terms of assessing model fit. We therefore followed the recommendations of Cheung and Rensvold,^30^ who found that the performance of the CFI can be considered to be independent of model parameters and sample size when testing for measurement invariance, and used a change in the CFI ≤ –0.01 as our criterion for invariance. Based on this assessment, we conclude that web and paper-based assessments of the DTDS possess measurement invariance.

An important limitation of our study was the lack of random assignment to the web- versus paper-based survey formats. In an attempt to address this limitation, we conducted the DIF analyses with covariates related to individual demographics (age and gender) as well as family socio-economic status (self reported family financial situation, mother’s and father’s highest level of education) and came to the same conclusion (i.e. there was no DIF present). Although it was not feasible to include the covariates in our latent variable models of measurement invariance, we were reassured that the results of the DIF did not change with the addition of the socio-demographic covariates. Although the results apply to our versions of the DTDS and the specific population of adolescents who completed the surveys, in a supervised classroom setting, it is reassuring to observe the instrument withstand this strict test of measurement invariance.

During the development and implementation of the BCYSOSH2, we found that school administrators strongly encouraged the development of web-based survey research methods that avoided the use of school staff to administer paper surveys or to install survey software on computers, minimized the schools’ involvement in the collection and distribution of confidential data, and ultimately reduced the amount of class time needed to facilitate survey research. Although many schools may be able to accommodate the completion of web-based surveys in supervised classroom settings, school administrators have indicated that they would ultimately like researchers to develop a system in which students complete web-based surveys on their own time either at home or in general use school computer labs. We believe that the results of this study begin to address this challenge by contributing to the ongoing validation of the DTDS as a tool for investigating the emergence of tobacco dependence among youth using both web- and paper-based survey methods and support the extension of our research to examine the effect of location of web survey administration (e.g. classroom versus home setting) in our future research on adolescent tobacco use.

## Figures and Tables

**Table 1:** Dimensions of tobacco dependence scale items.

Social dependence
S1. Smoking helps me fit in at school. S2. Smoking makes me feel popular. S3. Smoking makes me look cool. S4. Giving cigarettes to my friends makes me feel important. S5. Sharing cigarettes helps me feel closer to other people. S6. Smoking makes me look more mature. **Emotional dependence** E1. I need to smoke when I am stressed. E2. I need to smoke when I am sad or depressed. E3. I need to smoke to relax. E4. I need a cigarette to calm me down when I am angry. E5. I need to smoke when I am nervous. **Physical dependence** N1. I need to smoke in between classes. N2. I need to keep my nicotine levels up. N3. My smoking is automatic—I don’t even think about it. N4. I make sure I have enough cigarettes to get me through the day. N5. My body needs cigarettes to feel right. N6. I can function better after my first cigarette of the day. N7. My body craves cigarettes when I don’t smoke. N8. I like to smoke after I eat. N9. I feel panicked when I run out of cigarettes. N10. If I don’t have a cigarette, I don’t know what to do with my hands. N11. I smoke when I am alone. N12. Even when I don’t have time for a whole cigarette, I manage to fit in a few drags. N13. I can concentrate better after a cigarette. N14. I need to smoke when I am bored. N15. I find myself looking forward to my next cigarette. N16. My smoking follows a routine. N17. I spend a lot of time getting cigarettes. N18. I run out of cigarettes quicker than I think I will. N19. I have strong cravings to smoke cigarettes. **Sensory dependence** SE1. I like the feeling of blowing out smoke. SE2. Smoking makes things like having a pop or a coffee more enjoyable. SE3. I like the taste of cigarettes. SE4. I enjoy holding and handling cigarettes. SE5. I enjoy the feeling of smoke in my lungs.

**Table 2:** Demographic and smoking characteristics for web- and paper-based format groups.

Sample characteristic	Web (n = 354)	Paper (n = 1130)
**Age in years**		
Mean (SD)	16.1 (1.4)	15.9 (1.7)
**Gender (%)**		
Female	50.5	55.3
Male	49.5	44.7
**Family financial situation (%)**		
Poor	4.5	1.7
Below average	5.5	4.9
A little below average	10.7	9.6
Average	27.2	28.8
A little above average	19.0	20.6
Well-off	24.8	24.2
Very well-off	8.3	10.2
**Mother’s education (%)**		
Less than high school	12.4	20.5
Completed high school	48.8	41.1
Completed a post-secondary degree	38.8	38.4
**Father’s education (%)**		
Less than high school	26.7	27.4
Completed high school	30.9	30.3
Completed a post-secondary degree	42.4	42.3
**Lifetime number of cigarettes smoked (%)**		
A puff or a few puffs	17.3	3.9
1–5	9.5	8.2
6–15	12.1	9.3
16–25	6.9	8.3
26–99	11.8	14.1
More than 100	42.4	56.2
**Mean DTDS dimension scores (SD)**		
Social	10.6 (4.4)	11.0 (3.7)
Emotional	10.0 (4.9)	11.9 (4.8)
Physical	33.5 (15.7)	37.3 (15.7)
Sensory	11.4 (3.9)	12.3 (3.1)
**Cronbach’s alpha for DTDS scales**		
Social	0.93	0.90
Emotional	0.94	0.91
Physical	0.97	0.97
Sensory	0.85	0.78

**Table 3:** A comparison of web- versus paper-based responses to questions about survey comprehension, length and accuracy of responses.

Survey rating*	Web (n = 354)	Paper (n = 1130)
**Self reported accuracy of responses (%)**		
Very accurate	42.5	47.3
Mostly accurate	49.0	47.2
Mostly inaccurate	4.6	3.2
Very inaccurate	3.8	2.3
**Self reported comprehension (%)**		
I understood all the questions	68.2	66.0
I had difficulty understanding a few questions	28.0	31.6
I had difficulty understanding many of the questions	3.8	2.3
**Perceived length of questionnaire (%)**		
Much too long	40.5	38.5
A bit too long	37.1	40.4
About right	20.5	19.5
A bit too short	0.4	0.8
Much too short	1.5	0.8

* In reference to the entire questionnaire.

**Table 4:** Multigroup structural equation model tests of web- versus paper-based format measurement invariance.

Invariance model	Model constraints^a^,^b^	Model χ^2^ (df)^c^	CFI	RMSEA (90% CI)	SRMR
Configural	Equivalent configuration	4226 (1108)	0.920	0.063 (0.061, 0.065)	0.043
Metric	Equivalent loadings	4264 (1139)	0.920	0.062 (0.060, 0.064)	0.046
Scalar	Equivalent loadings and intercepts	4390 (1170)	0.917	0.064 (0.060, 0.064)	0.047
Error Variance	Equivalent loadings, intercepts and residual variances	4669 (1205)	0.911	0.064 (0.062, 0.065)	0.048

a Constraints refer to parameters estimated simultaneously in web- and paper-based format groups’ measurement models.

b To identify the models, the factor loadings of the first item in each dimension were fixed to equal 1.0 and the intercepts of these ‘marker’ items were constrained to be equal for the web- and paper-based format groups. Latent means of the web-based format group’s factors were set to zero as a referent.^19^

c All *p*-values are significant (*p <* 0.01).
